# Does Angling Technique Selectively Target Fishes Based on Their Behavioural Type?

**DOI:** 10.1371/journal.pone.0135848

**Published:** 2015-08-18

**Authors:** Alexander D. M. Wilson, Jacob W. Brownscombe, Brittany Sullivan, Sofia Jain-Schlaepfer, Steven J. Cooke

**Affiliations:** 1 School of Life and Environmental Sciences, Centre for Integrative Ecology, Deakin University, 75 Pigdons Road, Waurn Ponds, Victoria, 3216, Australia; 2 Fish Ecology and Conservation Physiology Laboratory, Dept. of Biology and Institute of Environmental Science, Carleton University, 1125 Colonel By Dr., Ottawa, ON, K1S 5B6, Canada; University of Regina, CANADA

## Abstract

Recently, there has been growing recognition that fish harvesting practices can have important impacts on the phenotypic distributions and diversity of natural populations through a phenomenon known as fisheries-induced evolution. Here we experimentally show that two common recreational angling techniques (active crank baits versus passive soft plastics) differentially target wild largemouth bass (*Micropterus salmoides*) and rock bass (*Ambloplites rupestris*) based on variation in their behavioural tendencies. Fish were first angled in the wild using both techniques and then brought back to the laboratory and tested for individual-level differences in common estimates of personality (refuge emergence, flight-initiation-distance, latency-to-recapture and with a net, and general activity) in an in-lake experimental arena. We found that different angling techniques appear to selectively target these species based on their boldness (as characterized by refuge emergence, a standard measure of boldness in fishes) but not other assays of personality. We also observed that body size was independently a significant predictor of personality in both species, though this varied between traits and species. Our results suggest a context-dependency for vulnerability to capture relative to behaviour in these fish species. Ascertaining the selective pressures angling practices exert on natural populations is an important area of fisheries research with significant implications for ecology, evolution, and resource management.

## Introduction

In recent years there has been a dramatic increase in research interest directed towards understanding the ecological and evolutionary implications of anthropogenic selection on natural populations [1, 2]. Of particular significance to aquatic biologists is the notion of harvest- or fisheries-induced evolution (FIE) [3, 4]. Selection in this context arises through selective harvesting (i.e., fishing mortality) associated with commercial or recreational fishing practices that can impact life-history traits (e.g. reduced adult body size, early sexual maturation) either directly, through decreased intrinsic growth, or indirectly through correlated behavioural traits [3, 5]. Depending on what gear is being used, both passive and directed harvesting techniques might disproportionately target fish based on their behavioural attributes. For example, gear that operate passively (gill nets) and depend on fish movement [4] as well as directed harvesting techniques such as those found in recreational angling might both preferentially catch specific behavioural phenotypes (e.g. bold/timid [6]). A common prediction is that individuals that tend to be more active, exploratory and willing to take risks are more susceptible to capture [7]. However, this is not necessarily always the case and may depend on such factors as methodology, target species and life-history stage [6, 8, 9]. Some studies have also suggested that individual-level behavioural characteristics as represented by an animal’s ‘personality’ or ‘behavioural type’ [10, 11], can be more indicative of an animal’s likelihood of capture/harvest than traditionally thought of morphological attributes alone [7, 12]. In this context, behavioural type refers to individuals who maintain consistent differences in their rank-order of behaviour for a particular attribute (e.g. aggression) despite environmental changes [11, 13].

Personality, an umbrella term for wide-spread consistency in behaviour across time and/or situations (also known as behavioural syndromes when suites of traits co-vary with one another), is thought to be important for ecology and evolution as it is often related to components of every animal’s daily behavioural repertoire including mating, foraging and locomotion [14]. Similarly, personality traits can also be heritable and are known to covary with physiological and life-history traits [15–17]. Should personality actually be related to individual capture probabilities and anthropogenic selection, then the widespread removal of specific individuals based on their behavioural type might result in the depletion of important genotypes/phenotypes from natural populations [10, 18]. Indeed, Philipp et al. [19] reported that vulnerability to recreational fisheries capture is a heritable trait among largemouth bass (*Micropterus salmoides*). Despite the implications and consequences of this notion for fisheries management and conservation practitioners [10, 11, 20–23], studies on this subject from a recreational fisheries perspective are still surprisingly scarce ([but see [6, 8, 24, 25]) and none have yet attempted to specifically assess the relationships between different artificial bait/lure varieties and behavioural types in wild fish. Artificial lure types, in particular, are ideal for testing many of the hypotheses associated with differential capture vulnerability as their presentation and techniques vary in terms of colour (natural and muted to un-natural and bright), retrieval rate (slow to fast) and disturbance (motion paths, noise, vibration) in the water column and are not confounded by chemical cues, odours of prey or food sources present in baited lures. In theory, such lures may vary in their level of selectivity for individuals based on aforementioned differences in behavioural type (bold versus timid).

**Fig 1:**
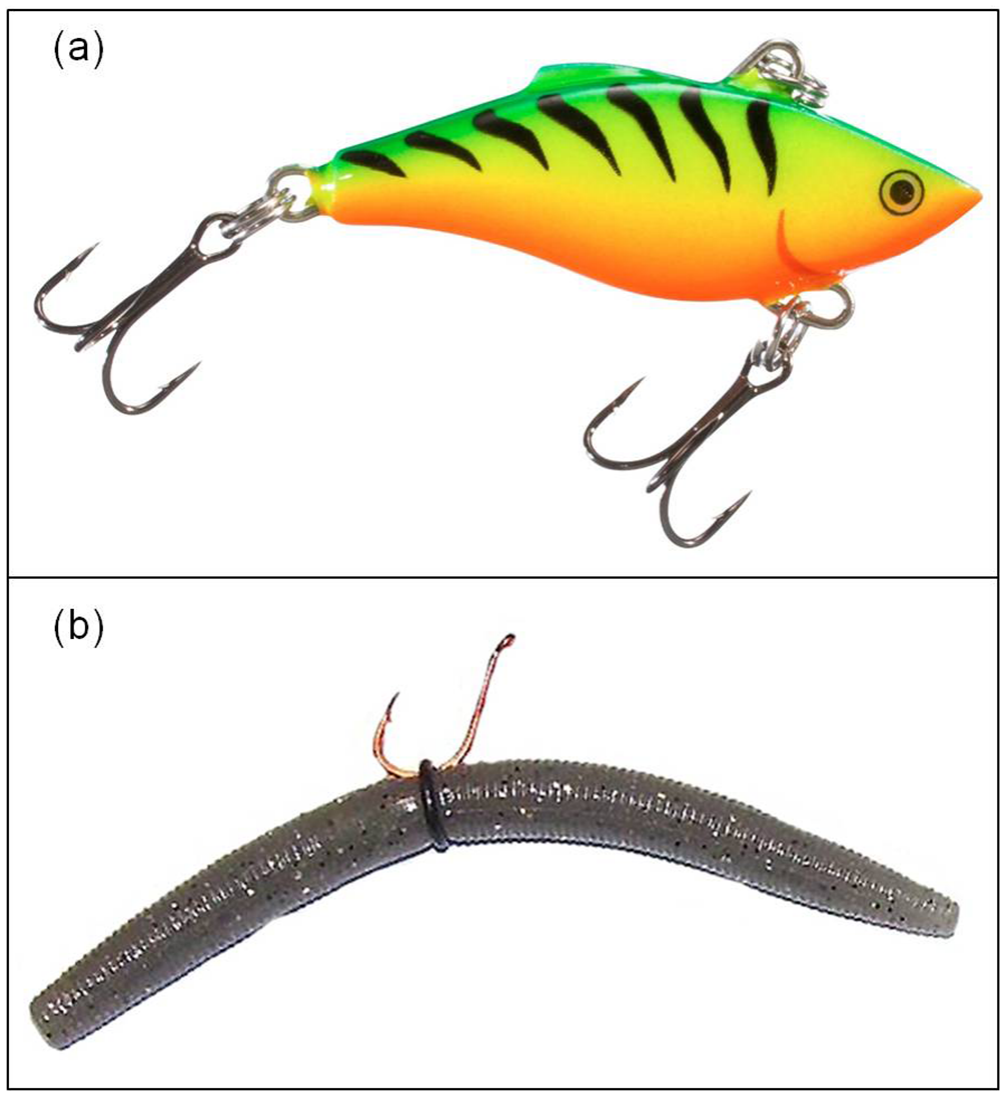
Lure types consisting of crank baits (a) and ‘wacky-rigged’ soft plastic baits (b) used to catch largemouth bass and rock bass in the wild. Crank baits included both diving lures and lipless surface lures equipped with rattles from several manufacturers and varied in colour, retrieve technique as well as level of water disturbance and motion paths (representative example: Rapala, Rattlin’ Rapala “fire tiger”, image credit: bassproshop.com). Soft plastic baits included a range of naturally coloured worms from two manufacturers and were allowed to sink and retrieved slowly (representative example: Gary Yamamoto Custom Baits-Yamasenko “pumpkin”, image credit: modified from heartlandoutdoors.com).

Here we tested whether or not two common recreational angling techniques and associated lure types (actively retrieved crank baits versus soft plastic worms fished more passively, Fig 1) differentially selected (and thus captured) fish based on their behavioural phenotype (bold/shy). Centrarchids are amenable to this research as they can be caught using a variety of angling methods, are abundant in many freshwater systems in North America, and are known to exhibit extensive individual-level differences in boldness [26, 27, 28]. Further, Centrarchids are also extremely popular sport fish for recreational anglers and are of interest to fisheries managers as many species are subjected to heavy angling pressures [21–23, 29–31]. We used largemouth bass (*Micropterus salmoides*) and rock bass (*Ambloplites rupestris*) across multiple life-history stages, caught from a large north temperate lake in Ontario, Canada. Our choices of species were based on the facts that both largemouth bass and rock bass inhabit similar environments, are popular sport fishes, and that our chosen angling techniques commonly capture these fishes. Should recreational angling target individuals based on their behavioural type in these species, we predicted that bolder more active fish would be caught more frequently using more actively retrieved, flashy, noisy hard plastic crank baits, while fish caught on more passively retrieved, natural-looking soft plastic worms would be more timid on average. This prediction is based on the assumption that while both bold and timid fish would be caught using natural-looking plastic worms, only bold individuals on average would be caught using the flashy crank baits. Ascertaining the relationship between angling practices and personality might have important implications for understanding the ecological/evolutionary consequences of selective harvest on wild fish populations and should therefore be of interest to evolutionary biologists as well as fisheries managers.

## Materials and Methods

### Field collections

Two different angling methods were used to collect 100 rock bass (total length, 180–275 mm [mean = 205 mm]; mass, 50–345 g [mean = 168 g]) and 100 largemouth bass (total length, 166–470 mm [mean = 301 mm]; mass, 68–1470 g [mean = 454 g]) from numerous locations comprising similar habitats (i.e. shallow bays with open water with weed cover below) in Lake Opinicon, Ontario, Canada (44°33′56.0” N, 76°19′23.6” W) between 3 July and 14 August 2014. Lake Opinicon was chosen as Centrarchids are subject to extensive recreational angling pressure year round and is therefore ideal for the purposes of our study. In-lake capture locations were separated by hundreds of meters to kilometres and were such that the breadth of this large lake was sampled, ensuring an accurate representation of the lake populations. Water temperatures were high yet stable during this summer study period (range: 24–26°C). Equal numbers of each species were caught using each of the two angling techniques (n = 50/technique/species), brought back to the laboratory and tested for individual level differences in standard metrics of personality [11, 14]; namely refuge emergence time [32, 33], flight-initiation-distance (FID) [34], general activity [35] and latency-to-recapture with a net [36] in the test arena. For both methods, angling was conducted using 2-m-long, medium-strength fishing rods and reels equipped with 4.5 kg break-strength, braided fishing line, which is typical gear for anglers targeting these species. For method one, terminal tackle included several different manufacturers’ brightly coloured diving or lipless 5–7 cm crank baits equipped with two size 4 treble hooks that were fished actively (Fig 1). These crank baits exhibited variable motion paths in the water and/or contained ‘rattles’ and were also capable of alternately floating and diving rapidly or ‘wiggling’ near the surface when retrieved. For method two, terminal tackle included a 1/0 circle hook, baited with a naturally coloured (e.g. leech [brown-green], pumpkin [brown], watermelon seed [dark green], wasp [dark red] or June bug [purple- black]) 12.7 cm ‘wacky-rigged’ plastic worm that was fished passively (Fig 1). Plastic baits were ‘wacky-rigged’ with a simple hook and O-ring, and in contrast to cranks baits, were silent (no rattles and sunk gradually when not being retrieved) and fished more passively (slow retrieval).

When a fish strike was detected (using either method)by the angler, the hook was set into the fish’s mouth as per typical angling events and the fish was landed as quickly as possible either by hand or using a rubberized fishing net. Our protocol was such that we aimed to have fish de-hooked and recovering in large coolers (containing fresh lake water, 45–90 litre) within ~45 s of initial strike to minimize fish stress from angling or air exposure. When possible, hooks were removed underwater and air exposure did not exceed 20 seconds, a duration which is deemed to be minimally stressful for these species [37, 38]. Fish that were deeply hooked or bleeding were not used in the study and immediately released. All fishing for both species occurred simultaneously, in the same areas and habitat types (though some unobservable variation in micro-habitats might exist between species/ sampling sites) and using the same lures to avoid a sampling bias associated with location of capture, habitat characteristics (submergent cover, rocks, emergent vegetation etc.) or lure type outside of our initial predictions. Fish that were caught using either of the two angling methods were held in separately marked coolers that had regular water exchanges prior to being brought back to the laboratory, where they were held in similarly marked holding tanks (see below). All fish were individually marked each day by clipping a small amount (<5mm) of one of the first 4 dorsal spines on the dorsal fin. Sampling (fishing) occurred everyday during the experimental period between the hours of 8am and 8pm. Once the daily maximum of 8 test fish was caught (of either species), sampling for that given day ceased. In general, largemouth bass were 2-3x more likely to be caught using either angling method than rock bass, which likely has to do with species abundance in the given sampling areas.

### Holding conditions and experimental arena

Following capture, fish were transferred to laboratory facilities at the Queen’s University Biological Station, located along the shoreline of Lake Opinicon. Upon arrival fish were held in one of two large holding tanks (max. 4 fish per 2 m diameter tank, water depth 50 cm, 1 for fish caught via one of the two angling methods) for 24 hrs prior to behavioural testing. Holding tanks were allocated randomly each day to avoid any potential tank effects between groups. This holding period is a standard unit of time frequently used in fish behaviour studies as it is long enough for gut contents to be evacuated but not long for feeding to be required, thus ensuring standardized conditions among focal individuals [6, 26]. Holding tanks were aerated and provided with fresh lake water via a flow-through system. Given the short holding time in the laboratory (< 30 hours) and the potential risk of introducing additional state-dependent changes in behaviour associated with individual differences in willingness to feed in the laboratory after capture, we did not feed fish prior to testing. That said, since all fish were caught initially via a feeding attempt (lure grab), it is likely safe to assume all fish were interested in doing so to some extent, and thus in a similar hunger state.

**Fig 2:**
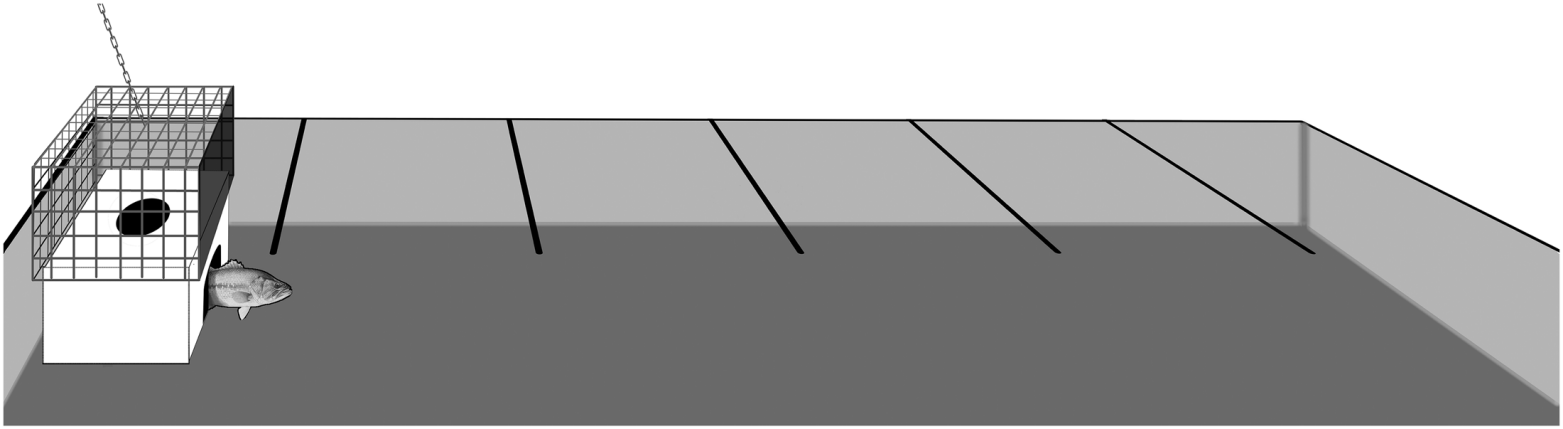
Schematic representation of the in-lake experimental arena tank used to quantify aspects of largemouth bass and rock bass personality including refuge emergence time, flight-initiation-distance, general activity and latency-to-recapture with a net.

All behavioural testing took place in a large, *in situ* (in lake), natural experimental arena consisting of a converted boat slot (2.6 x 6 m) (Fig 2). The experimental arena was located within a covered boat house which provided ample indirect natural and artificial lighting. Observation blinds consisting of black plastic were installed on all four sides of the arena to prevent disturbance from passers-by, and included small windows (30 x 30 cm) to allow for observation and data collection. Beneath the water surface the arena was surrounded by cement walls on three sides and by a series of mesh screens on the outer wall to allow water circulation but prevent fish escape or entry of other non-test fish or debris. Water temperature was identical to holding tanks due to continuous flow through circulation of lake water during holding. The arena also contained an acclimation chamber on one side, which consisted of an opaque plastic box and a slightly larger outer box made of steel mesh. The inner box included an opening on the dorsal surface to allow the addition of a test fish prior to the onset of a trial as well as second opening on side of the box to allow the fish to exit during behavioural testing. The outer box consisted entirely of steel mesh (to facilitate removal without disturbing the inner box) except for one outer edge that was covered in plastic and covered the exit opening on the inner box. The base of the arena was covered uniformly in sand, rocks and leafy detritus to simulate natural conditions. Lastly, the surface of the arena was divided into 6 regions by 5 lines of string which were tied across the width of the arena at 1 m intervals (Fig 2). These lines were meant to facilitate later measurements of individual-level differences in activity and exploration.

### Behavioural trials

Approximately 24 h after capture, each focal fish was gently caught using a rubberized net, placed in a small cooler and transferred to the acclimation chamber of the experimental arena. The focal fish was then given 15 min to acclimatize prior to the onset of behavioural testing. After acclimation, the outer mesh box was lifted manually from behind the blind, therein allowing the focal fish to exit the chamber and swim freely throughout the experimental arena (Fig 2). Each focal fish was given 20 min to exit the acclimation chamber. Any individuals that did not exit were assigned the maximum possible value (20 min), whereupon the chamber was raised via a remote pulley system and fish were given an additional 10 min to acclimate prior to further testing. After exiting, several behavioural measures were quantified for each focal fish including latency-to-exit the chamber, time spent active, FID, and latency-to-recapture with a net in the test arena. Time spent active was quantified as the total number of 1 m arena sections the fish crossed over a period of 10 min. Individual differences in FID were quantified as the minimum distance necessary to initiate a flight response from a standard dip-net. Sedentary focal individuals were approached at an approximately fixed angle and rate of approach along the longitudinal axis, beginning 1.5 m from the test fish. Latency-to-recapture was quantified as the time required to capture the focal fish using a black rubberized fishing net (50 cm diameter) using a standardized rate of approach. These traits (refuge emergence and activity in particular) were chosen as they have been shown to be highly consistent and repeatable in terms of estimating personality attributes in Centrarchids and indeed within every study that has sought to test this within a sampling season in this taxon [26–28, 39, 40].

At the completion of behavioural trials, all focal fish were released into a nearby bay in Lake Opinicon, which was not proximal to any other sampling location in the lake. The caudal fin of each focal fish was also fin-clipped (a small section of fin was removed) to further ensure no individuals were tested twice in the event of a recapture. That said, no individuals were recaptured during our study, likely due to the high abundance of these species in the lake and the distance between sampling locations.

### Data analyses

The latency-to-exit-refuge metric was analyzed with Cox proportional hazards regression (a time- to-event analysis) with species, lure type, fish total length, and interactions between species and lure type as well as lure type and total length as predictors. The assumption of proportional hazards was checked prior to analysis. This analysis was used because not all fish exited the refuge within the 20-minute test period, and therefore the data were censored. For all other metrics, a series of generalized linear models (JMP v7.0) were run with species, lure type (crank bait versus soft plastic), body size (total length) and interaction terms between lure type*body size, species*lure type and species*body size as predictors, and personality metrics (activity, FID & latency to recapture) as response variables. When appropriate, data were log transformed to meet necessary statistical assumptions.

#### Behavioural correlations and Syndrome analysis

Comparisons of individual behavioural traits (refuge emergence, flight-initiation-distance, latency-to-recapture, general activity) and body size were calculated using Spearman’s rank correlation tests. To avoid an inflated chance of Type 1 error, our alpha level for this analysis was adjusted to be more conservative using the sequential Bonferroni correction.

To test for the potential effects of a boldness syndrome on angling vulnerability we also collapsed individual traits representing risk-taking behaviour (refuge emergence, flight-initiation-distance and latency-to-recapture) into a first principal components score using Principal Components Analysis (PCA) (Table 1). Across-context correlations with activity, angling technique as well as body size were then calculated using Spearman’s rank correlation tests. To avoid an inflated chance of Type 1 error, our alpha levels for this analysis was adjusted to be more conservative using the sequential Bonferroni correction.

**Table 1:** PCA loadings of within-context behavioural variables used to generate a first principal component score (PC1) for boldness in rock bass and largemouth bass.

Behavioural Context	Behaviours within each context	Loadings for PC1	% Variation explained
Boldness	Refuge emergence	-0.76	41.1
	Flight-initiation-distance	0.80	
	Latency-to-recapture	0.11	

### Ethics statement

This study was approved by the institutional Animal Care Committee at Carleton University (protocol 101051) and thus adheres to the guidelines of the Canadian Council on Animal Care and the laws of Canada. All animals were released immediately into Lake Opinicon after their participation in the study.

## Results

**Fig 3:**
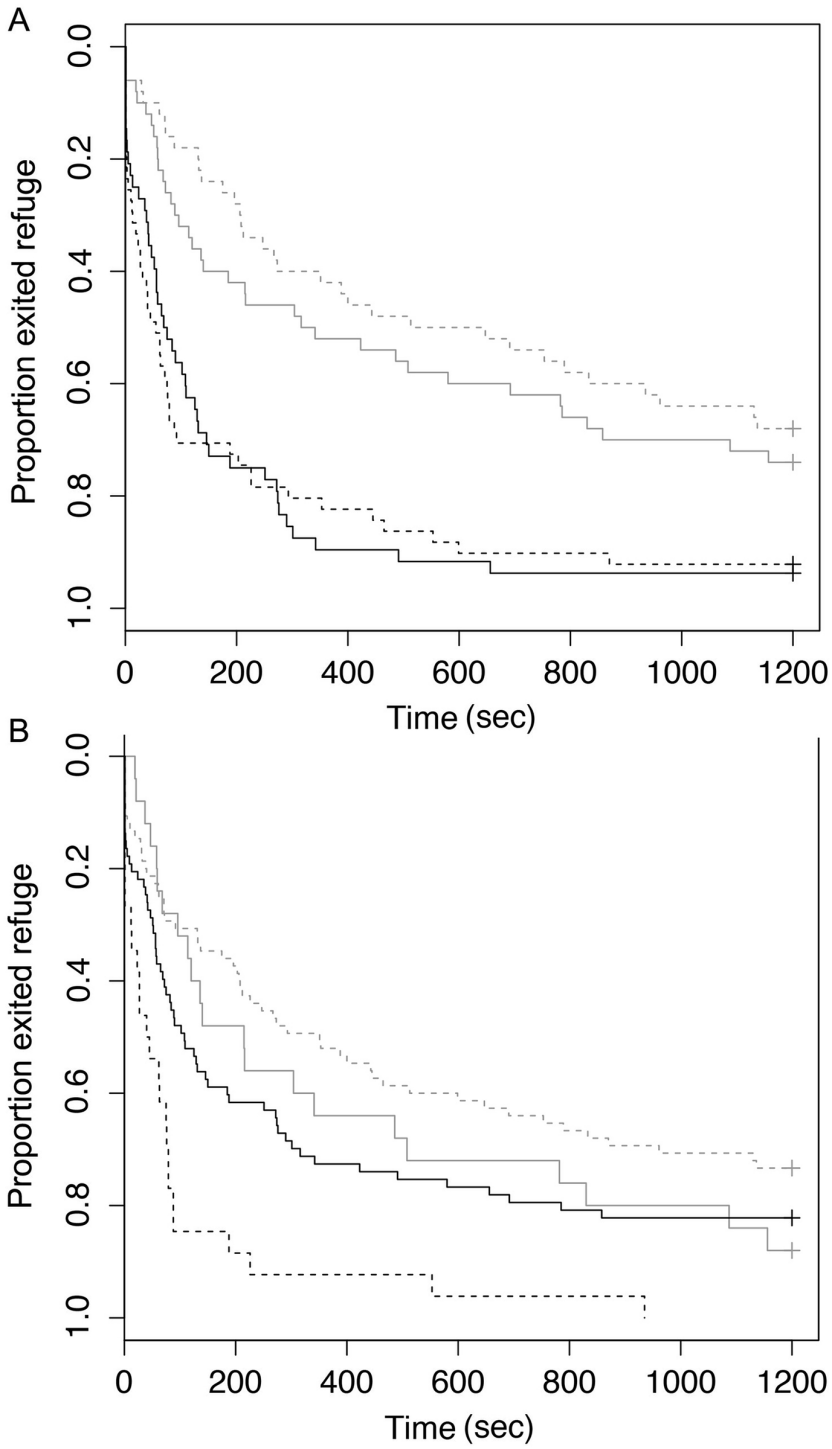
Proportion of fish exiting refuge (latency-to-exit) during behaviour trials for (A) Largemouth Bass (grey) and Rock Bass (black) caught with crank baits (solid lines) and soft plastic baits (dashed lines) as well as (B) proportion of large (>20cm Rock Bass, >30cm Largemouth Bass; grey) and small fish (<20cm Rock Bass, <30cm Largemouth Bass; black) caught with crank baits (solid lines) and soft plastic baits (dashed lines) that exited the refuge.

There were significant effects of species, lure type, and interaction between lure type and body size (total length) on latency-to-exit refuge (Table 2). Rock bass exited refuge faster than largemouth bass. In general, fish caught on crank baits exited refuge faster than those caught on soft plastic baits in both species. However rock bass that had the shortest latency to exit times initially demonstrated an opposite trend, though this difference was not observed in individuals with longer latencies (Fig 3A, Tables 2 and 3). Small fish (within each species) tended to exit refuge faster than large fish (Fig 3B). Species was also a significant predictor of activity and latency to recapture with largemouth bass more active and avoiding net capture significantly longer than rock bass on average (Tables 2 and 4). Similarly, neither lure type, nor the interactions between lure type, species and body size were significant predictors of any behavioural metrics other than latency-to-exit refuge (Tables 3 and 4).

**Table 2:** Output from Cox proportional hazard regression for latency-to-exit refuge for Largemouth Bass and Rock Bass captured via angling using crank baits or soft plastic baits.

Factor	Coefficient	Standard error	z	p-value
Species	**1.09**	0.29	3.76	*<0.001*
Lure type	**2.61**	0.91	2.86	*0.004*
Body size	**0.02**	0.02	1.09	0.28
Species x Lure type	**-0.63**	-0.40	1.57	0.12
Lure type x Body size	**-0.09**	-0.03	3.11	*0*.*002*

**Table 3:** Output from mixed models of Largemouth Bass and Rock bass behavioural metrics by lure type and body size (Total Length). The table shows the response metric, the model terms, F- and P-value. Significant model terms are shown as italicized (α = 0.5).

Metric	Model Terms	Parameter Estimates	F-value	P-value
Activity	Species	4.23	*9*.*89*	*0*.*002*
	Lure type	0.65	0.60	0.441
	Body size	0.21	0.98	0.324
	Species x Lure type	-0.47	0.17	0.680
	Lure type x Body size	0.22	2.09	0.150
	Species x Body size	0.22	1.14	0.288
Flight-initiation-distance	Species	-4.24	.38	0.242
	Lure type	-1.47	0.43	0.515
	Body size	-0.54	0.93	0.334
	Species x Lure type	0.26	0.008	0.930
	Lure type x Body size	0.02	0.002	0.963
	Species x Body size	-0.45	0.65	0.421
Latency-to-capture (net)	Species	17.45	*6.37*	*0.048*
	Lure type	-8.86	2.57	0.109
	Body size	-0.46	0.46	0.734
	Species x Lure type	-3.37	0.23	0.647
	Lure type x Body size	0.49	0.27	0.616
	Species x Body size	-0.28	0.04	0.837

**Table 4:** Summary of means ± standard errors for raw values of behavioural and morphological attributes of Largemouth Bass and Rock Bass used in the current study. All traits listed are significantly different between species (F = 7.24–156.1; P < 0.008).

Behavioural / Morphological attribute	Species	Mean ± SE
Latency-to-exit (s)	LB	583.4 ± 48.7
	RB	193.6 ± 31.8
Activity (# line crosses)	LB	19.2 ± 1.5
	RB	8.9 ± 0.7
Flight-initiation-distance (cm)	LB	30.5 ± 2.9
	RB	44.8 ± 3.3
Latency-to-capture (s)	LB	53.5 ± 9.4
	RB	24.7 ± 5.1
Total length (cm)	LB	30.9 ± 0.74
	RB	20.5 ± 0.40
Mass (g)	LB	453.1 ± 30.4
	RB	165.6 ± 7.3

### Behavioural correlations and Syndrome analysis

When considered independently, body size and most behavioural attributes were not significantly correlated with each other using the sequential Bonferroni adjusted alpha levels. The only exception to this trend was that more active largemouth bass also took longer to be recaptured (*rs* = 0.2823, P = 0.004)

Spearman rank correlation tests between the boldness PC1 score (representing refuge emergence, flight-initiation-distance and latency-to-recapture) as well as activity, body size and angling technique revealed significant across-context correlations between individual boldness score and activity in both largemouth bass (*rs* = 0.317, P = 0.001) and rock bass (*rs* = 0.288, P = 0.004), but not with body size or angling technique (P > 0.05). Suggesting a generalized boldness-activity syndrome is not related to angling in this context.

## Discussion

In line with our initial predictions, the fishing lures and techniques associated with our study do appear to selectively target largemouth bass and rock bass based on refuge emergence, a standard measure of assessment for boldness in fish, but this effect is not reflected in a boldness syndrome incorporating other behavioural attributes overall. While flight initiation distance, activity, and latency-to-recapture were not significantly different between fish captured on the two lure types, latency-to-exit refuge was significantly higher in large fish caught on more natural, passively fished plastic worms than fish caught on actively fished, flashy, noisy hard plastic lures. This relationship however, in more complicated in that while the trend is retained among small fish caught on cranks baits, small fish caught on worms appeared boldest overall, suggesting size is an important consideration in future work. Refuge emergence is considered a standard measure of assessment for boldness in fishes (see [41, 42, 43] for select examples), with bolder fish typically exiting refuge faster than more timid fish. This supports our prediction that lure type influences selectivity for behavioural traits in these fish species.

We also observed strong differences between species in most personality metrics, where largemouth bass tended to take longer to exit refuge, yet were generally more active and evaded recapture longer than rock bass. There were also effects of body size on personality measures, where larger fish tended to take longer to exit refuge. While rock bass were generally smaller than largemouth bass, this trend held within species as well. Interestingly, in contrast to our initial predictions, we also found that small fish that were caught on worms exited faster on average (Fig 3B) than larger fish or small fish caught on crank baits. Based on this initial data set, it is difficult to speculate as to the underlying bases for this difference, but our results do suggest a context-dependency for vulnerability to capture relative to personality in these species and highlight body size as an important factor in need for further investigation. There are a number of potential selective pressures that could act differentially on fish behavioural tendencies across life stages (e.g. predation risk, competition, angling selectivity).

A number of recent studies have suggested that bolder, more active and exploratory fish should be most vulnerable to angling due to presumed associations with higher growth rates and metabolic needs (i.e. higher foraging rates) [3, 7, 44]. However, actual experimental evidence of this notion remains mixed particularly when considering differences between domesticated or captive reared fish and wild ones. For example, Klefoth et al. [45] found that domestication resulted in certain strains of common carp (*Cyprinus carpio* L.) being bolder in terms of foraging tendency; resulting in an increased vulnerability to angling. Similarly, Härkönen et al. [24] found that individual based differences in activity (used as a proxy for exploratory tendency) in hatchery-reared brown trout (*Salmo trutta*) were linked to angling vulnerability, perhaps due to underlying differences in state-dependent factors (i.e. hunger) associated with higher growth rates. Collectively, these studies suggest that angling vulnerability might be related to varying metabolic needs associated with higher growth rates that could, in turn, be indicative of different behavioural types. That said, other studies looking at two lines of largemouth bass artificially selected for high- and low-vulnerability to angling [see 19] found differences in foraging ecology and prey capture success [46], but not routine locomotory activity [47] despite documented differences in metabolism [25, 44] which, in turn, could suggest that such relationships are not clear cut and complex in nature.

An additional complication arises when the few studies examining angling selectivity on wild fish *in situ* are considered. Indeed, supportive evidence is deficient or even at times contradictory to the increased vulnerability hypothesis. For example, Kekäläinen et al. [8] used two different simple lure types while ice-fishing (natural and artificially baited hooks) for European perch (*Perca fluviatilis*) and found that boldness did not correlate with lure type nor capture order. Similarly, Wilson et al. (2011) found that angling (using simple baited hooks) in the wild appeared to target timid rather than bold juvenile bluegill sunfish (*Lepomis macrochirus*). Wilson and colleagues suggested that personality-based vulnerability to angling might vary by life-history stage, habitat, species and particular angling technique/ gear being used [6], which might explain why it is difficult to reach a general consensus (particularly between laboratory and field studies). As a result of this early study’s predictions, we tested whether these two different lure types and angling techniques would differentially impact individuals (across life-history stages) in two species based on their behaviour. Our findings, while preliminary, are the first to support this vulnerability to capture hypothesis in wild fish and provide a platform considering the ecological consequences of commonly used recreational angling techniques. However, additional research needs to be done to confirm and/or further elucidate these relationships following similar methodologies.

A potential limitation of our approach is that angling in general might be biased towards a particular behavioural type (i.e. bold or timid), as such we might not be capturing a representative subset of the entire population [12], as very bold or timid individuals may never or rarely be caught using these techniques. However, this notion is not overly relevant for the purposes of the current study. Our experimental design was directed towards understanding if two popular fishing techniques (and associated lures) used by recreational fishers’ differentially targeted fishes based on their behaviour attributes. Further, our approach of using multiple lure types within each category/technique provides the most comprehensive exploration of this phenomenon to date. In addition, by avoiding the confounding influence of natural baits (i.e. chemical cues) our techniques were entirely reliant on the properties of the angling approaches themselves, and might in part also explain why previous studies on wild fish have observed different trends [8, 48], though these studies also varied in numerous other factors (season, species, technique etc.).

There is little doubt that the relationship between fish personality traits (e.g. boldness) and susceptibility to recreational and commercial harvesting practices is important for behavioural ecologists and fisheries managers. Indeed, there is growing general concern that harvesting practices, including those found in recreational angling, are causing fisheries-induced evolution by artificially selecting fish that have certain behavioural and physiological traits. Even in recreational fisheries where many fish are released (as is the case for both largemouth bass and rock bass; [49, 50]), post-release mortality can at times be high (reviewed in [51]) which is functionally analogous to harvest (i.e., both are fishing mortality). Moreover, different fishing techniques may be used by anglers with different levels of expertise and harvest modalities (i.e., conservation-oriented behaviors; [52]). Given that lure types and hook configurations influence hooking injury and even likelihood of mortality [51, 53], it is conceivable that FIE could occur independent of harvest. From the standpoint of FIE, it is also important to establish whether the mechanism underlying harvest vulnerability has a heritable component or is based on developmental factors (ie. experience). While we did not assess this possibility in the current study, previous studies on personality and capture susceptibility do suggest it is a likely possibility [7, 47].

It is apparent that anthropogenic selection in the form of recreational fishing mortality can alter the phenotypic composition of natural populations over time, which can be detrimental in certain contexts [19, 25]. Here, we provide preliminary evidence that lure types and associated angling techniques can influence selectivity for wild fish with certain behavioural types (at least in terms of their refuge use), where more active fishing methods generally selected for bolder fish. While it is possible that other capture techniques, species and populations will exhibit different relationships and trends in behaviour than those described here, our results do suggest that this effect in a recreational context is significant and certainly an area of research concern from a fisheries management standpoint [21, 23, 31].
